# Principles of mental health care during the COVID-19 pandemic

**DOI:** 10.1192/j.eurpsy.2020.54

**Published:** 2020-05-20

**Authors:** Martina Rojnic Kuzman, Marko Curkovic, Danuta Wasserman

**Affiliations:** 1 Department of Psychiatry, Zagreb University Hospital Centre, Zagreb, Croatia; 2 University of Zagreb, School of Medicine, Zagreb, Croatia; 3 University Psychiatric Hospital Vrapce, Zagreb, Croatia; 4 Karolinska Institutet, National Centre for Suicide Research and Prevention of Mental Ill-Health, Stockholm, Sweden

**Keywords:** COVID-19, mental health, organization, public health, stigma

## Abstract

We describe the basic principles of mental health care during the COVID-19 pandemic that should be endorsed by the mental health professional associations and incorporated in the health strategies for the management of the COVID-19 pandemic. The main principle is that there should be no substantial differences in the provision of health care for COVID-19 between persons with pre-existing mental health disorders and the ones without previous disorders. Subsequently, the organization of the health care should reflect that as well. These principles should (a) prevent the possible effects of stigmatizing attitudes toward mental health issues, possibly leading to potentially deleterious situations, such as psychiatric patients being treated (even temporarily) separately from other patients, in psychiatric facilities, where the staff is not equipped and trained adequately for the management of COVID-19; (b) highlight the fact that patients with mental health disorders are at greater risk for developing serious complications of COVID-19 infection due to other factors—they often smoke and have comorbidities such as hypertension, diabetes, all associated with higher morbidity and mortality from COVID-19 infection; (c) highlight that measures should be taken to minimize the risk of the spread of infection in psychiatric wards/institutions; (d) provide a general framework for the reorganization of mental health services toward the provision of services for persons in need, including frontline medical workers and patients with COVID-19 without previous mental health problems as well as for persons with pre-existing mental health problems under new circumstances of pandemic.

The World Health Organization (WHO) developed following a series of events, with the first one of them being the International Sanitary Conference (in 1851), organized as an attempt of the Western countries to stop the spreading of cholera by maritime routes from Asia.

Subsequently, series of activities were introduced to limit the epidemics and set the ground for achieving the global health, including the revision of the international lists of diseases and causes of death, prevention of malaria, biological standardization, control of tuberculosis, quarantine, unification of pharmacopeias, control of sexually transmitted diseases, international pandemic control, revision of the pilgrimage clauses, international control of habit-forming drugs, alcoholism, crime prevention, housing and town-planning, influenza, insulin, medical examination of immigrants, public health service, and radiotherapy in uterine cancer [1,2]. Laying down the principles of health as having socioeconomic, political, and cultural dimensions, and thus organizing the health care based on the principles of holism and availability to everyone, these pivotal decisions of the WHO granted the same standard of care to all persons, not excluding persons with mental disorders and disabilities [2]. Subsequently, all major professional and ethical principles granted all people with equal right to health care, opposing against any kind of discrimination, including because of mental disorders or disabilities. However, despite this progress, a high level of stigma associated with mental health still exists worldwide. Persons with mental disorders, their families, and sometimes even their carers, are historically subjected to stigma and suffer from the consequences of the stigmatizing behavior of the society, including social isolation, poorer chances for employment, but also decreased access to health care [3].

With the situation of the pandemic, the rise in stigma toward the vulnerable population or the population perceived as the source of danger is significant and may have deleterious consequences [4]. More specifically, this attitude may result in inadequate health care for persons with mental illness, and lead to devastating consequences. For example, during the outbreak of the corona virus in China, as well as in South Korea, a high number of psychiatric patients quickly become infected with SARS-CoV-2, while the number of psychiatrists as well as the equipment, was generally lacking, which eventually led to the reorganization of the psychiatric care [5,6].

Thus, it is crucial that we learn from lessons from the past and assure that there is an equal level of health care for persons with mental disorders. While the WHO clearly stated that the management of mental disorders is listed among the essential health services to be guarantee during the COVID-19 pandemic [7], it is essential that the organization of mental health services during the COVID-19 pandemic is supported uniformly by mental health professionals, to ensure its adequate implementation. We propose several basic principles for the organization of mental health care, as follows:

1. There should be no substantial differences in the treatment of SARS-CoV-2 infection between persons with mental disorders and those without mental disorders. The same care for physical health and safety should be available and provided to persons with pre-existing mental disorders and their close ones as to all other members of society. Concordantly, mental health services and staff should receive the personal protective equipment under the same epidemiologically justified conditions as other health care services.
2. Health care policy makers and planners should envisage the unintended secondary consequences of the widely applied restrictive measures on non-pandemic related health conditions and illnesses as well as on various other socioeconomic factors alongside beneficial effects on controlling the pandemic, and ensure adequate resources to tackle the issues emerging from this unprecedented interruptions of social structures and functions. Having in mind the influences of social determinants of health, this may be of particular importance in the aftermath of COVID-19 pandemic. Here, of special importance are mental health disorders as not only are they generally prevalent, but may also affect previously, in that sense, presumably healthy populations, such as health care workers and other frontline responders. The pandemic context followed by comprehensive restrictive public health measures (such as isolation, quarantining and monitoring) may cause significant distress resulting in occurrence of mental disruptions and disorders in general, and of previously unaffected populations [6].
3. Considering that the necessary restrictions implemented as security measures for the containment of the infection during the pandemic with SARS-CoV-2 also increase the risk of low adherence and interruption of long-term pharmacological treatments and of rehabilitative interventions of patients with mental health disorders, it is critical to ensure a rearrangement of psychiatric services in order to maintain the continuation of mental health care to all persons with pre-existing mental disorders. This may include several interventions: (a) organization of available (24/7) psychiatric liaison service in health care facilities for patients infected with SARS-CoV-2, on-line or in person, respecting all safety measures; (b) organization of available (24/7) on-line psychiatric liaison service in health care facilities for patients suspected of infection with SARS-CoV-2 or close contacts requiring isolation at home; (c) organization of available (24/7) psychiatric services in psychiatric facilities for patients with pre-existing mental health problems, on-line or in person, respecting all safety measures. These services should employ a proactive strategy in ensuring adherence to previous mental health treatment by actively contacting the patients, and ensuring support thought remote (phone/on-line/digital) services and ensuring services that allow the maintenance of previous pharmacotherapy (especially long acting psychiatric medication or substitution therapy for patients with addiction problems). This may imply establishing a closer collaboration with general practitioners and pharmacists, as well as the introduction of e-medical documentation and remote (phone lines/on-line/digital) psychiatric services if there were not any.
4. Persons with pre-existing mental disorders, but also persons with no known previous mental disorders may develop safety/life threatening behavior (such as agitation, suicidal behavior or aggression) during the infection with SARS-CoV-2. Agitation can also develop as a symptom of delirium, as part of the COVID-19 pneumonia, but also as a symptom of the abstinence from psychoactive drugs during isolation and/or during more focused physical treatment. These conditions, even though those usually require immediate psychiatric care and interventions should not be considered as an absolute indication for the admission to psychiatric facilities, especially if these are not adequately prepared for physical care. Instead, mental health services should be widely available as a consultation service, and urgent mental health care should be delivered in a separate unit of the same facilities where somatic care is provided, only for the duration of the imminent threat.
5. It is necessary to employ all preventive measures to limit the spread of pandemics in psychiatric facilities. Population of patients with chronic mental health conditions present a group of persons particularly vulnerable for development of complicated COVID-19 illness, due to the presence of firmly established mortality risk factors, including smoking, comorbidities such as metabolic syndrome, hypertension, especially if they are of older age [8]. In combination with the lack of adequate care and safety for the SARS-CoV-2 infection and the lack of trained specialists, persons in these facilities may become especially disadvantaged. If a person with mental illness is suspected/diagnosed with COVID-19, they should be transferred to the same facilities where persons with no mental illness are treated, following the same epidemiological and somatic triage principles. Mental health care should be continued and delivered by extensively established mental health consultation services.
6. Mental health care should be organized following principles of holism, availability, and accessibility to patients and medical staff, according to good clinical practice and epidemiological principles. Secondly, it should be organized following the principles of collaboration with all other providers of mental health services. In that sense, there is a great need to adjust mental health services to be widely available to the most severely affected populations: (a) persons with suspected or with developed SARS-CoV-2 infection with psychiatric symptoms; (b) front line medical staff; (c) persons with pre-existing mental health problems; (d) all others which lives, health, and safety is severely affected by the pandemic and corresponding public health measures [9]. As pointed previously, the delivery of services is provided mostly through remote services. However, given the possible issues of novelty (and therefore issues with coverage and utility) and various issues inherent to these methods (such as security and privacy) “traditional” mental health services should be also available, especially to those in greatest need, respecting all epidemiological safety measures [10]. As COVID-19 pandemic context may create significant burdens on health care systems and therefore create a need for rationing and prioritization of health care resources, there is a great need to anticipate, develop, and apply necessary guidelines in that sense, as leaving this kind of decisions to frontline caregivers is not only unethical, but creates a substantial and significant additional burden with far reaching consequences [7].
7. The term “social distancing” which is being widely used by government officials, media, and the public evokes associations of being rejected, isolated, and distanced, particularly among vulnerable groups such as those with mental disorders. For this reason, the technically correct term of “physical distancing” should be adopted and used instead of “social distancing” by all government officials, media, and the general public [11]. It is a distance of 2 m apart that will reduce the spread of the virus, not social distancing. It is paramount that inclusive language is used during these difficult times.
8. Professionals working in the field of mental health should be actively involved in the organization of the health care by providing education, guidance, and administrative framework/supporting documents to disseminate relevant information to policy makers, health care workers, and general public. Here they should not only serve as advocators for their patients (persons with mental health disturbances and disorders), but may contribute significantly to anticipating the needs, planning, and delivering much needed mental health protection of the whole community.

Exceptional times urge for exceptional measures. However, the burden of exceptional measures, if applied unwisely, tend to fall on the shoulders of those most vulnerable, further increasing already existing inequalities and inequities. Every strategy and measure of battling the pandemic has its’ efficacy, efficiency, and safety profile. The public health ethics stance should be promotion and provision of equality for all persons and just distribution of harms and benefits within a society. In that sense, advancement of societies could be measured on how their members treat the most vulnerable among them, not only during peaceful and prosperous times, but also, or even more indicatively, during demanding times marked by widespread and omnipresent uncertainties.

## Data Availability

No additional data are available in relation to this manuscript.
